# Different interface orientations of pentacene and PTCDA induce different degrees of disorder

**DOI:** 10.1186/1556-276X-7-248

**Published:** 2012-05-14

**Authors:** Angela Poschlad, Velimir Meded, Robert Maul, Wolfgang Wenzel

**Affiliations:** 1 Steinbuch Centre for Computing, Karlsruhe Institute of Technology (KIT), Karlsruhe, 76131, Germany; 2 Institute for Nanotechnology, Karlsruhe Institute of Technology (KIT), Karlsruhe, 76131, Germany

**Keywords:** Organic interfaces, Organic electronic devices, Interface disorder, Molecular dynamics, PTCDA, Pentacene

## Abstract

Organic polymers or crystals are commonly used in manufacturing of today‘s electronically functional devices (OLEDs, organic solar cells, etc). Understanding their morphology in general and at the interface in particular is of paramount importance. Proper knowledge of molecular orientation at interfaces is essential for predicting optoelectronic properties such as exciton diffusion length, charge carrier mobility, and molecular quadrupole moments. Two promising candidates are pentacene and 3,4:9,10-perylenetetracarboxylic dianhydride (PTCDA). Different orientations of pentacene on PTCDA have been investigated using an atomistic molecular dynamics approach. Here, we show that the degree of disorder at the interface depends largely on the crystal orientation and that more ordered interfaces generally suffer from large vacancy formation.

## Background

Organic light emitting diodes (OLEDs), organic solar cells, organic thin films transistors, etc. are made of organic polymers or crystals [1-3]. The effect of the disorder in organic devices on optoelectronic properties was analyzed by Rim et al. [4]. They showed an increased photocurrent generation with improved molecular order. It occurs due to the influence of the stacking on the exciton diffusion length. Hu et al. measured a strong dependence of the conductance across highly oriented pentacene nanocrystals on the packing orientation [5]. The influence of packing on charge transport in organic solids was also analyzed using Monte Carlo methods [6]. Kwiatkowski et al. [6] were able to predict the mobilities of electron and holes for ordered and disordered Alq3. Different functional organic materials were reviewed by Ishii et al. [7]. They highlighted the energy level alignment and electronic structures at organic/inorganic and organic/organic interfaces of, for example, Alq3, 3,4:9,10-perylenetetracarboxylic dianhydride (PTCDA) and 1,4,5,8-tetrathiafulvalene (TTF).

**Figure 1:**
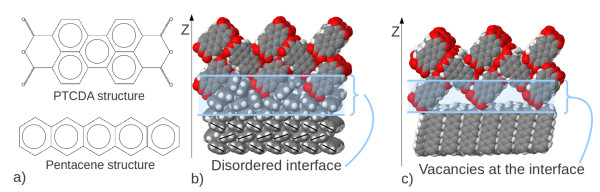
**Pentacene and PTCDA: Chemical formulas and interface formation.** (**a**) Chemical formulas of PTCDA (top) and pentacene (bottom) are presented. (**b**) Example of the realistic interface formed by PTCDA (212) on pentacene (100). After full MD relaxation cycle, pentacene molecules have closed the holes coming from the zigzag nature of the PTCDA surface in a disordered fashion. (**c**) Example of the realistic interface formed by PTCDA (212) on pentacene (001). After full MD relaxation cycle, pentacene molecules remain in crystalline structure forming vacancies coming from the zigzag nature of the PTCDA surface.

In our work, the morphology of interfaces between pentacene [8] and PTCDA [9] was analyzed (Figure 1a). Both molecules form different crystal modifications. Pentacene is known to have a high temperature (HT) and a low temperature (LT) polymorph. Yoneya et al. [8] showed that the LT polymorph is destabilized by substrates and transforms into HT polymorph. Therefore, the HT polymorph was used as the base for simulations. For PTCDA, the *α*polymorph [9] was used.

Molecular orientation at interfaces is decisive for predicting optoelectronic properties such as exciton diffusion length [10], charge carrier mobility [11], and molecular quadrupole moments [12]. Verlaak et al. analyzed the impact of the molecular quadrupole moments, influenced by e. g., material and crystal orientation on the interface energetics. An insight on models of electronic processes across organic interfaces is given by Beljonne et al. [13], while a review of the corresponding theoretical approaches is presented by Brédas [14].

Our study of organic-organic pentacene/PDCDA interfaces is organized as follows: after a brief introduction presented above, we proceed with the presentation of the methods followed by the results and some conclusive remarks.

## Methods

The molecular dynamic (MD) simulations of the interfaces between PTCDA and pentacene have been performed with the atomistic molecular dynamics package GROMACS (Stockholm Center for Biomembrane Research, Stockholm, Sweden and Biomedical Centre, Uppsala, Sweden) [15] using the generalized amber force field (GAFF) parameterization [16] for organic molecules, having Yoneya et al.’s work [8] in mind, and ESP charges [17] calculated with the semi-empirical quantum chemistry package MOPAC (Stewart Computational Chemistry, Colorado Springs, CO, USA) [18]. The parameter conversion from amber to GROMACS was done with the help of Antechamber python parser interface (ACPYPE) [19], the recommended tool for using GAFF with GROMACS, cf [8,20-22]. After simulation, a check of basic molecule parameters was done and the results for the example of pentacene are presented in Table 1. A more detailed report on relative errors in energy, dehidrals, etc can be found in the ACPYPE wiki [23].

**Table 1:** Comparison of calculated and experimental relevant parameters

**Bonds in pentacene**										
	**C1-C2**	**C2-C3**	**C3-C4**	**C4-C5**	**C5-C6**	**C6-C7**	**C4-C21**	**C6-C19**	**C-H**	
*Ab initio*	1.43	1.38	1.44	1.4	1.42	1.43	1.46	1.46	1.1	
Exp	1.441	1.358	1.428	1.381	1.409	1.396	1.453	1.464	na	
MD	1.397	1.394	1.395	1.399	1.395	1.395	1.403	1.403	1.088	
**Angles in pentacene**										
	**C-C-C**	**C-C-C**	**C-C-C**	**C-C-C**	**C-C-C**	**C-C-C**	**C-C-C**	**C-C-C**	**C-C-C**	**C-C-C**
Exp	121	123	124	124	123	119	119	118	118	118
MD	120.2	120.1	120.1	120.3	120.2	119.8	119.8	119.9	119.8	119.8

A representative selection of bonds and angles of pentacene is presented. A comparison of measured [24] and *ab initio*[25] parameters with results from the MD calculations is displayed showing a generally good agreement between the methods.

The systems were simulated with a step size of 0.5 fs for more than 3 ns at a temperature of 300 K using a Berendsen thermostat [26] for temperature control. The van der Waals cut-off was set to 1.2 nm, the Coulomb cut-off to 5 nm and the relative permittivity was set to four which was taken from Wang et al. [27]. No periodic boundary conditions were used owing to the different crystal lattices.

**Figure 2:**
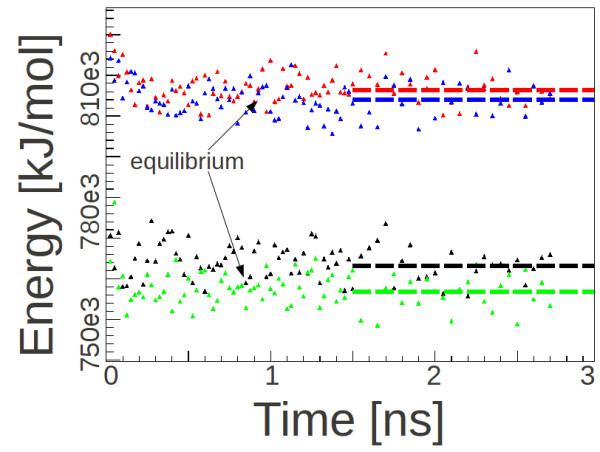
**Time-evolution of mean energy per molecule for the four interfaces of (-221) PTCDA and (100) pentacene.** The triangles mark the mean energy at subsequent time steps where each relative orientation is represented by a different color. After few hundred picoseconds, equilibrium is reached and the energy, driven by the given temperature, fluctuates around an average value. The dashed lines represent the average energy between 1.5 ns and 3 ns of simulation time.

Three surfaces were chosen and combined. For pentacene the surfaces (100), (010), and (001) were used and for PTCDA the surfaces used are (102), (-221), and (212) as defined by Miller indices. The combination of these surfaces led to nine different interface facets, e. g., (212) on (010) and (-221) on (001), as depicted in Figure 1b,c showing their relaxed structures, leaving rotation and translation as degrees of freedom. An optimal relative orientation within each of these nine facets was found by performing four simulations each with relative orientations from being twisted against each other. After a total energy comparison, the structure with the lowest mean energy per molecule of the fully relaxed systems was chosen. As an example, the energy-evolution for the interface facet (-221)//(100) is shown in Figure 2. The set of simulations were done on systems arranged to fill a 10 × 10 × 10 nm^3^ cube with each crystal type, filling half the space.

## Results and discussion

**Figure 3:**
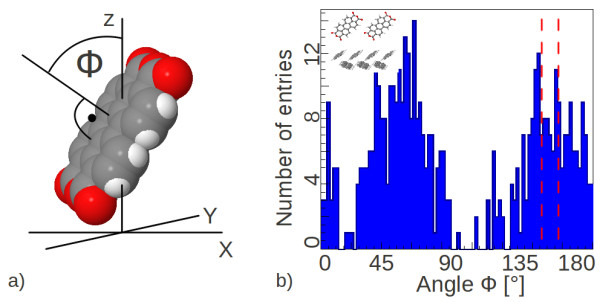
**Definition of the angle defining the molecular orientation along with a distribution at one interface.** (**a**) *ϕ*is the angle between the normal of the molecule plane and the normal of the interface plane (z-axis), i. e. the angle between the molecule plane and the interface plane. (**b**) Distribution of the angle *ϕ*(blue area) for the relaxed pentacene molecules at the interface of (-221)//(100) (pictogram in the left upper corner). Contributing pentacene molecules have a PTCDA neighbor with maximal atom-atom distance of 0.4 nm. The red dashed lines at 145.5 degree and 157 degree mark the values for the ideal morphology.

In order to quantify the disorder at the pentacene/PTCDA interface, we used distribution of *ϕ*, defined as the angle between the molecular and the interface plane (or rather their respective normals) as shown in Figure 3. Owing to the fact that the molecules will start to relax, they will start to deviate from the bulk values. The more molecules have different *ϕ*, the more disordered is the structure.

**Figure 4:**
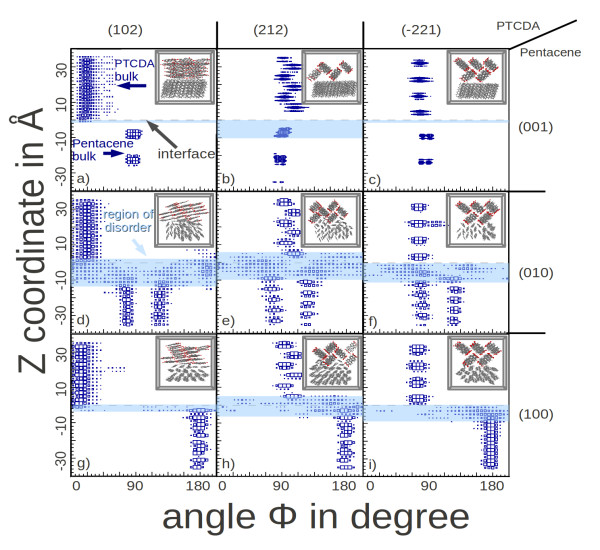
**Angular distribution of angle***ϕ***as function of distance from the interface.** The 2d-histograms (**a–i**) show the angle *ϕ*depending on the distance to the interface at *z*=0, where *ϕ*is the angle between the molecule plane and the interface plane (see Figure 3a). The distance to the interface is given as the *z* coordinate of the molecule center in Å. Each histogram represents the results for one of the interface facets configurations given in Miller indices (001), (010), and (100) for pentacene (as marked on the right side of the histograms) and as (102), (212) and (-221) for PTCDA (as marked above the histograms). The box size is proportional to the number of occurrence. The interface location is emphasized by a dashed line with PTCDA located above and pentacene below it. The region of disorder is marked in light blue. Outside the light blue area the crystals are in their bulk phase. The corresponding relaxed crystal morphology is represented by the inset molecular structure.

In the histograms of Figure 4, the *y*-axis was defined as distance in Å from the (ideal) interface in *z*-direction, while the *x*-axis shows the angle distribution. Light blue regions mark the disordered regions. Two clear patterns can be observed: 1) size of the disordered region can vary from 2 to 16 Å, and 2) the disorder seems to spread asymmetrically from the ideal interface, clearly preferring pentacene-rich regions. The first pattern can be explained as having two competing effects at the interface, one being the optimization of the intermolecular distance/interaction and the other being the conservation of bulk properties. The second pattern can be understood in the light of much stronger *Π*−*Π* stacking of the PTCDA molecules, leading to a stronger intermolecular interactions, and greater energies are required to disrupt these molecules from their bulk positions when compared to pentacene bulk.

## Conclusions

Analysis of PTCDA/pentacene interfaces was performed with two emerging messages: there seems to be two competing effects, one coming from intermolecular interaction, which leads to disordered interfaces, while the other coming from the preservation of bulk properties results in large interfacial vacancies. Both of the effects would lead to dramatically diminished transport properties. Namely, increased disorder would cause greater energy disorder of the interfacial hopping sites, while interfacial vacancies would lead to diminished intermolecular overlaps, or hopping matrix elements. Whether which of the competing effects is influencing more the hopping transport properties is the focus of our ongoing research. Our second observation is that pentacene seems to be, in general, a more flexible material, which can be observed from the fact that the disordered regions are predominantly pentacene-rich.

## Competing interests

The authors declare that they have no competing interests.

## Author’s contributions

AP carried out the molecular dynamics calculations, the setup of the initial system and helped in drafting of the manuscript, and revisions. VM helped in analysis and interpretation of data, and drafted the manuscript and revisions. RM provided the calculation of the partial charges. WW participated in the design of the study, formulated the original scientific question and helped in analysis and interpretation of data. All authors read and approved the final manuscript.

## Authors’ information

AP is Ph.D. student, VM and RM have Ph.D. degree in physics, and WW is an associate professor at Karlsruhe Institute of Technology.
